# Reliability of the IDENTIFY calculator in stratifying risk of urothelial carcinoma in patients with haematuria: An initial evaluation at an Australian centre

**DOI:** 10.1002/bco2.291

**Published:** 2023-09-09

**Authors:** Paul M. Rival, Lucas Ooi, Hyun Lim, Anusha Varughese, Monika Pandey, Caroline Dowling, Shomik Sengupta

**Affiliations:** ^1^ Department of Urology Eastern Health Melbourne Victoria Australia; ^2^ Eastern Health Clinical School, Faculty of Medicine, Nursing and Health Sciences Monash University Melbourne Victoria Australia

**Keywords:** bladder cancer, haematuria, model validation, risk stratification, urothelial carcinoma

In 2020, there were 3330 new cases of bladder cancer in Australia; 90% were urothelial cancer (UC).^1^ Both non‐visible haematuria (NVH) and visible haematuria (VH) can indicate the presence of bladder cancer, with VH conferring two to four times the risk.^2^ There are no official Australian guidelines for the investigation of haematuria, but experts recommend urine cytology, a CT intravenous pyelography (IVP) or renal ultrasound and specialist referral for cystoscopy for patients with VH.^3^ For NVH, they recommend low‐risk patients have repeat microscopy in 6 months and to consider urine cytology, imaging and referral for cystoscopy for higher risk patients.

The NICE guidelines^4^ recommend referral within 2 weeks for non‐urinary tract infection (UTI) VH in patients over the age of 45 and NVH in patients over the age of 60 with either dysuria or raised white blood cell (WCC) on bloods, with no requirement for urine cytology or imaging. The AUA/SUFU guidelines^5^ recommend upper tract imaging, urine cytology and referral for cystoscopy for all patients with VH. For those with NVH, they recommend risk stratification, with repeat urinalysis in 6 months for low‐risk patients, renal ultrasound and referral for cystoscopy for intermediate‐risk patients and CT/MRI upper tract imaging and referral for cystoscopy for high‐risk patients, with a recommendation to avoid urine cytology.

Even though it is recognised across international guidelines that patients with VH are at higher risk and require more investigation, to date, there is no standardised approach to risk stratifying patients and no consensus on the need for cytology, the need for imaging at time of referral or its modality, nor the need for or urgency of cystoscopy. This suggests further research is required to establish an optimal diagnostic pathway for haematuria that would enhance the detection of UC while minimising patient risk and resource wastage. The IDENTIFY calculator,^6^ developed in 2022 based on multicentre international data collection,^7^ provides an online tool that uses patient characteristics such as age, gender, past medical history, smoking exposure and presence of specific symptoms and signs to calculate a risk score for developing urothelial cancer (UC).

We conducted a retrospective single‐centre observational cohort study from October 2021 to September 2022 to audit how we investigated and managed patients referred with haematuria and assess the potential effectiveness of the IDENTIFY calculator as an adjunct to the clinical decision‐making process. Ethics approval was obtained from the Office of Research and Ethics at Eastern Health. Patients newly referred to the clinic with haematuria were included in the study if they had no history of previous urological malignancy. Patient characteristics, previous medical history and the results of investigations as part of the haematuria workup were collected from medical records.

Eighty‐nine patients were included in our study. Patient characteristics, time to cystoscopy as well as results of investigations are summarised in Table 1. Most referrals were from primary care (70%), with 24% from the emergency department and the rest from other specialties. Two‐thirds of patients were referred with VH, 62% were male, and 75% were over the age of 50. The majority of patients were investigated with urine cytology (69%), CT IVP (57%) and cystoscopy (78%, with 9% having a biopsy). It took a median of 3 weeks for patients to have their cystoscopy after the initial review in clinic.

**TABLE 1:** Summary of findings for patients newly referred to the urology department with haematuria.

	Comparison by diagnosis of UC		Total	Comparison by IDENTIFY risk stratification group			
	Patients ultimately diagnosed with UC	Patients ultimately NOT diagnosed with UC		Patients stratified to high‐risk group (IPR > 20%)	Patients stratified to intermediate‐risk group (IPR 5%–20%)	Patients stratified to low‐risk group (IPR 1%–5%)	Patients stratified to very‐low‐risk group (IPR < 1%)
Patient characteristics							
Number of patients (%)	5 (6)	84 (94)	89 (100)	29 (33)	34 (38)	17 (19)	9 (10)
IPR: median and (range)	40.1 (17 to 60.6)	9.3 (0.07 to 54.5)	9.7 (0.07 to 60.6)	33.4 (20.1 to 60.6)	9.5 (5.8 to 19.1)	2.4 (1.1 to 4.7)	0.6 (0.07 to 0.9)
NVH (%)	2 (40)	27 (32)	29 (33)	3 (10)	9 (26)	9 (53)	8 (89)
VH (%)	3 (60)	57 (68)	60 (67)	26 (90)	25 (74)	8 (47)	1 (11)
Age: mean and (SD)	77.6 (10)	62.2 (17)	63 (17.1)	76.2 (9.4)	63.1 (15.1)	49.2 (14.8)	46.2 (12.6)
Male (%)	5 (100)	50 (60)	55 (62)	21 (72)	24 (71)	7 (41)	2 (22)
Female (%)	0 (0)	34 (40)	34 (38)	8 (28)	10 (29)	9 (53)	7 (78)
Investigations							
Number of patients investigated with **urine cytology** (%)	4 (80)	57 (68)	61 (69)	22 (76)	19 (56)	15 (88)	5 (56)
Number of patients where **urine cytology was suggestive of UC** (%)	3 (75)	1 (2)	4 (7)	4 (18)	0 (0)	0 (0)	0 (0)
Number of patients investigated with **CT IVP** (%)	4 (80)	47 (56)	51 (57)	23 (79)	17 (50)	8 (47)	3 (33)
Number of patients where **CT IVP was suggestive of UC** (%)	4 (100)	1 (2)	5 (10)	5 (22)	0 (0)	0 (0)	0 (0)
What were the relevant CT IVP findings?	Urinary bladder wall thickening VUJ mass with obstruction of UO (×2) Bladder filling defect	Abnormal renal pelvis and proximal ureter with suspicious TCC		Urinary bladder wall thickening VUJ mass, UO obstruction (×2) Bladder filling defect Abnormal renal pelvis and proximal ureter, suspicious TCC	—	—	—
Number of patients investigated with **cystoscopy** (%)	4 (80)	65 (77)	69 (78)	22 (76)	26 (76)	15 (88)	6 (67)
Time between clinic and cystoscopy (weeks)							
Mean	5.0	4.21	4.35	3.0	3.9	5.6	7.1
Median	5.0	3.0	3.0	3.1	2.3	6.0	3.2
SD	1.5	4.0	3.9	2.1	3.7	3.2	8.5
Number of patients investigated with a **biopsy at time of cystoscopy** (%)	4 (100)	2 (3)	6 (9)	5 (23)	1 (4)	0 (0)	0 (0)
Number of patients where **biopsy‐confirmed diagnosis of UC** (%)	4 (100)	0 (0)	4 (67)	3 (60)	1 (100)	—	—
What were the relevant biopsy results?	Non‐invasive low‐grade papillary UC (×3) High‐grade invasive UC (×1)	—		Non‐invasive low‐grade papillary UC (×2) High‐grade invasive UC (×1)	Non‐invasive low‐grade papillary UC (×1)	—	—
Did the patient receive a confirmed diagnosis of UC by CT IVP or biopsy?							
Yes (%)	5	—	5 (6)	4 (14)	1 (3)	0 (0)	0 (0)
No (%)	—	84	84 (94)	25 (86)	33 (97)	17 (100)	9 (100)

Of the 89 patients, five were diagnosed with bladder cancer, all male, with median age 73 years, with three referred for VH and 2 NVH, all painless. They had a high average smoking pack year history (median of 62.5), and two had a family history of UC. They mostly had urine cytology and CT IVP findings suggestive of UC and received a diagnosis of UC by biopsy following cystoscopy (except one patient who was diagnosed on CT IVP but was deemed too unwell to proceed with cystoscopy). It took a median of 5 weeks for these patients to get their cystoscopy.

The remaining 84 patients were a mix of males (60%) and females (40%), on average younger (median 64.5 years) and referred with a similar proportion of VH (68%). They had a lower average smoking pack year history (median of 18) and were more likely to have had UTI symptoms (18%), suprapubic pain (17%) and dysuria (23%) associated with haematuria. A majority were investigated with urine cytology (68%) and CT IVP (56%), while only 2% of each were suggestive of UC. A significant majority were investigated with cystoscopy (77%), but only 2% had a biopsy (negative for UC). It took a median of 3 weeks for these patients to have their cystoscopy.

These findings suggest that differentiating patients solely on VH and NVH appears insufficient for risk‐stratifying patients—in contrast to published data,^2^ the risks of bladder cancer were comparable between VH and NVH in our study. Additionally, there was no significant difference in time to cystoscopy comparing patients who were diagnosed with UC and those who were not, and a significant proportion of patients seem to be over‐investigated.

Therefore, we used the IDENTIFY calculator to investigate its reliability in risk stratifying this cohort of patients. For every patient, the IDENTIFY predicted risk (IPR) was calculated using the online calculator. The patients were stratified into four groups as recommended: very low risk (IPR < 1%), low risk (IPR of 1%–5%), intermediate risk (IPR of 5%–20%) and high risk (IPR > 20%).

The IDENTIFY calculator stratified all five patients diagnosed with UC as high (4/5) or intermediate (1/5) risk. No urine cytology or CT IVP findings were suggestive of UC in intermediate‐, low‐ and very‐low‐risk groups. No cystoscopies were suggestive of UC in low‐ and very‐low‐risk groups. Comparing each group for time till cystoscopy, we found significant differences between the Intermediate‐ and high‐risk groups (median wait‐time 2.3 and 3.1 weeks, respectively), compared to the low‐risk group (6 weeks).

Our findings suggest that, based on the IDENTIFY risk calculator, high‐ and intermediate‐risk patients could be prioritised to undergo urine cytology, CT‐IVP and urgent cystoscopy. Conversely, low‐ and very‐low‐risk patients could selectively consider omission of urine cytology, de‐escalation of imaging by way of renal ultrasound and lower urgency or selective cystoscopy, thus reducing costs, invasiveness and risk of investigations. Of note, numerous biomarkers have been developed to similarly guide haematuria investigations, although disappointingly to date none have shown sufficient utility to be incorporated into standard haematuria investigation pathways.^8^ Future assessment of the IDENTIFY calculator should include comparison and/or integration with existing and new biomarkers.

In conclusion, the IDENTIFY calculator shows promise as a standardised stratification tool in our cohort of patients' workup for haematuria. It appears more reliable than simply categorising patients into VH versus NVH groups and could potentially be useful to appropriately prioritise haematuria investigations as outlined above. Further research with a larger cohort, as ongoing from the IDENTIFY study group,^7^ is needed to confirm these findings and prospectively assess the use of this tool in clinical practice.

## AUTHOR CONTRIBUTIONS

*Conceptualisation*: Paul Rival. *Methodology*: Paul Rival, Caroline Dowling, Shomik Sengupta. *Validation*: Paul Rival, Shomik Sengupta. *Formal analysis*: Paul Rival. *Investigation/data collection*: Paul Rival, Lucas Ooi, Hyun Lim, Anusha Varughese, Monika Pandey. *Resources*: Caroline Dowling, Shomik Sengupta. *Data curation*: Paul Rival, Lucas Ooi, Hyun Lim, Anusha Varughese, Monika Pandey. *Writing—original draft preparation*: Paul Rival. *Writing—review and editing*: All co‐authors. *Visualisation*: Paul Rival, Shomik Sengupta. *Supervision*: Caroline Dowling, Shomik Sengupta. *Project administration*: Caroline Dowling, Shomik Sengupta.

## CONFLICT OF INTEREST STATEMENT

SS is a Board Director (honorary) of ANZUP trials group and has been a speaker or advisor for Mundipharma, Abbvie, Ipsen, Janssen, MSD and the Eastern Melbourne Primary Health Networks (all honoraria donated directly into institutional research fund). Other authors have no conflicts to declare.

## References

[1] Bladder cancer: causes, symptoms & treatments [Internet]. Cancer Council. [cited 2023 Apr 27]. Available at: https://www.cancer.org.au/cancer-information/types-of-cancer/bladder-cancer

[2] Khadra MH , Pickard RS , Charlton M , Powell PH , Neal DE . A prospective analysis of 1,930 patients with hematuria to evaluate current diagnostic practice. J Urol. 2000;163(2):524–527. 10.1016/S0022-5347(05)67916-5 PMID: 10647670. 10647670

[3] Assessment and management of haematuria [Internet]. Australian Journal of General Practice. [cited 2023 Apr 27]. Available at: https://www1.racgp.org.au/ajgp/2021/july/haematuria‐in‐the‐general‐practice‐setting

[4] Recommendations organised by site of cancer: suspected cancer: recognition and referral: guidance [Internet]. NICE. [cited 2023 Apr 27]. Available at: https://www.nice.org.uk/guidance/ng12/chapter/Recommendations‐organised‐by‐site‐of‐cancer#urological‐cancers

[5] Microhematuria: AUA/SUFU guideline (2020) ‐ American urological association. [cited 2023 Apr 27]. Available at: https://www.auanet.org/guidelines-and-quality/guidelines/microhematuria

[6] Burst [Internet]. [cited 2023 Aug 22]. Available at: https://www.bursturology.com/Studies/Identify/Admin/

[7] Khadhouri S , Gallagher KM , MacKenzie KR , et al. Developing a diagnostic multivariable prediction model for urinary tract cancer in patients referred with haematuria: results from the IDENTIFY collaborative study. Eur Urol Focus. 2022;8(6):1673–1682. 10.1016/j.euf.2022.06.001 35760722

[8] Sathianathen NJ , Butaney M , Weight CJ , Kumar R , Konety BR . Urinary biomarkers in the evaluation of primary hematuria: a systematic review and meta‐analysis. Bladder Cancer. 2018;4(4):353–363. 10.3233/BLC-180179 30417046 PMC6218111

